# A web application to support the coordination of reflexive, interpretative toxicology testing

**DOI:** 10.1016/j.jpi.2023.100303

**Published:** 2023-02-26

**Authors:** Abed Pablo, Thomas J. Laha, Nathan Breit, Noah G. Hoffman, Andrew N. Hoofnagle, Geoffrey S. Baird, Patrick C. Mathias

**Affiliations:** a Department of Laboratory Medicine and Pathology, University of Washington School of Medicine, Seattle, WA, USA; b Department of Medicine, University of Washington School of Medicine, Seattle, WA, USA; c Department of Biomedical Informatics and Medical Education, University of Washington School of Medicine, Seattle, WA, USA

**Keywords:** Python, Laboratory workflows, Custom web application, Quality control, Mass spectrometry, UDT, urine drug testing, LC-MS/MS, Liquid chromatography-tandem mass spectrometry, QC, Quality control, RRT, Relative retention time, AMR, analytical measurement range, S/N, Signal to noise ratio, GC-MS, gas chromatography-mass spectrometry, mg, milligram, MLS, medical laboratory scientist, LIS, laboratory information system, AWS, Amazon Web Services, S3, Simple storage service, TSV, tab-separated values, XML, Extensible markup language, CSV, Comma-separated values, UW Medicine, Department of Laboratory Medicine and Pathology at University of Washington Medicine

## Abstract

**Background:**

Reflexive laboratory testing workflows can improve the assessment of patients receiving pain medications chronically, but complex workflows requiring pathologist input and interpretation may not be well-supported by traditional laboratory information systems. In this work, we describe the development of a web application that improves the efficiency of pathologists and laboratory staff in delivering actionable toxicology results.

**Method:**

Before designing the application, we set out to understand the entire workflow including the laboratory workflow and pathologist review. Additionally, we gathered requirements and specifications from stakeholders. Finally, to assess the performance of the implementation of the application, we surveyed stakeholders and documented the approximate amount of time that is required in each step of the workflow.

**Results:**

A web-based application was chosen for the ease of access for users. Relevant clinical data was routinely received and displayed in the application. The workflows in the laboratory and during the interpretation process served as the basis of the user interface. With the addition of auto-filing software, the return on investment was significant. The laboratory saved the equivalent of one full-time employee in time by automating file management and result entry.

**Discussion:**

Implementation of a purpose-built application to support reflex and interpretation workflows in a clinical pathology practice has led to a significant improvement in laboratory efficiency. Custom- and purpose-built applications can help reduce staff burnout, reduce transcription errors, and allow staff to focus on more critical issues around quality.

## Background

Urine drug monitoring is an increasingly important tool for primary care physicians and pain specialists to monitor patients on chronic opioid therapy.^1^ Routine and random monitoring for all patients on long-term opioid therapy is recommended prior to starting and throughout drug therapy.^2^ There are various analytical methods and matrices available for patient monitoring. Urine drug testing (UDT) has become the “gold-standard” for detecting illicit drug use and monitoring ongoing therapy. Urine is non-invasive, readily available, and contains higher concentrations of drugs and metabolites compared with other matrices. Immunoassay testing, commonly referred to as urine drug screening, detects the presence of selected drugs and/or metabolites based on a detection threshold. Typically, the immunoassay functions as the initial evaluation for the potential detection of drugs but because it lacks specificity for analytes of the same drug class, it can lead to false-positive and -negative results. Therefore, drug screens are often coupled with a confirmatory detection method for more specific testing. Chromatography is generally reserved for confirmatory or definitive testing following immunoassay screening. Historically, gas chromatography-mass spectrometry (GC-MS) was the standard for confirmatory testing. However, liquid chromatography-tandem mass spectrometry (LC-MS/MS) has gained favor over GC-MS due to reduced complexity of sample preparation methods and the broader applicability of LC-MS/MS to different drug classes.^3^, ^4^

Many laboratories are equipped to support UDT.^1^^,^^3^, ^4^^,^^8^, ^9^ A major challenge of staged drug testing (i.e., screening and confirmatory assays), is the review and interpretation of the large number of quantitative and qualitative results that is generated by screening and confirmatory methods.^5^, ^6^, ^7^ Pharmacokinetic mechanisms dictate how quickly and how much of a drug and its metabolites appear in an individual's urine and can be challenging for physicians who review a limited number of these results to understand.^5^ An incorrect interpretation of the LC-MS/MS results could mistakenly suggest that a patient used a non-prescribed medication. Additionally, the potential for analytes to co-elute could affect the signal of the analytes, influencing the overall accuracy of the LC-MS/MS test.^4^ Yang et al.^9^ described that adding interpretative comments from a trained laboratory director to urine drug screening results can greatly impact both the clinician and patient. Clinicians may not understand the analytical method limitations and misinterpretation of the results may lead to a patient's inappropriate termination from care or stigmatization. The authors further reported an increase in urine drug screen orders with interpretive comments indicating that clinicians value the interpretation made by a trained director. Because accurate interpretation of results often requires the integration of documented clinical information, medication data, test results, pharmacokinetics, and test limitations and interferences, assigning a trained pathologist or clinical chemist who is familiar with interpreting clinical information as well as the biochemistry and testing methods to provide an interpretation of the results is a valuable tool in ensuring the correct diagnosis for patients.

One challenge laboratories and laboratory directors face with UDT is that there may be results from several tests that inform whether additional testing is required and need to be integrated to provide an interpretation given the specific clinical context. Within the Department of Laboratory Medicine and Pathology at University of Washington Medicine (UW Medicine), we have formulated a urine drug testing ordering protocol and interpretation workflow. Clinicians may select from a menu of panels that are based on the assessed patient's risk of treatment non-compliance. Panels include an immunoassay drug screen and confirmation LC-MS/MS assays for opioids, amphetamines, benzodiazepines, and alcohol. For example, a low-risk patient panel consists of an immunoassay drug screen, and aberrant or unexpected results can lead the reviewing pathologist to order one or more of the confirmation tests. On top of the interpretation challenges, turnaround times for the different tests are unevenly distributed. Most laboratory information systems are not equipped to track, collect, and display the different results in an effective and informative manner to support this workflow.

The aim of this project was to design and implement a web-based software application that centralizes test results for pathologist review, performs the quality control calculations for the complex LC-MS/MS analysis of opioids and their metabolites, functions as a user input form for pathologist interpretation entry, and auto-files results into the laboratory information system (LIS).

## Methods

The initial step before designing the application was to understand the entire workflow, then gather requirements and specifications from stakeholders.

### Pre-application testing and workflows

At UW Medicine, for each patient specimen undergoing the opiate confirmation assay, 2 extractions are prepared, an undiluted and a diluted 10-fold urine sample. To help improve the complex LC-MS/MS data analysis of opioids and opioid metabolites, a command-line software application was developed.^10^ The software application known as “SMACK” was shown to improve the data analysis process by automating a quality control algorithm and calculations to improve consistency of analysis as well as reducing the amount of time medical laboratory scientists (MLS) spent reviewing the data.

The algorithm was part of an intricate workflow that involved several steps, spreadsheets, and individuals. The goal of the spreadsheets was to collect and consolidate the relevant patient information so that the reviewing pathologist had the necessary information to generate an interpretation for the overall case. The information was spread across several spreadsheets with the relevant patient data which relied on laboratory staff and pathologists copy-and-pasting various fields from one spreadsheet to another. Pathologists and laboratory staff communicated the completion of steps via email and a naming convention for the relevant files.

### Requirements

We set to gather information on how clinicians ordered the different drug monitoring tests and how the information arrived at the laboratory. We investigated the LIS data workflow that was in place and how this information could feed the application including assay results and patient demographics. Additionally, we worked with the laboratory to understand how test results were produced and how this information should be included in the application. Lastly, we investigated what the requirements were for progressing from one step of the workflow to the next along with the data associated with the progression. In addition to the workflow in the laboratory, we needed to understand what data elements are needed for each step of the workflow. With this information, we would create a map of the workflow to visualize and better understand the workflow. We gathered necessary components from stakeholders, pathologists, directors, pathologists in training, and laboratory staff by interviewing stakeholders and observing workflows. During this process, we gathered user interface requirements from stakeholders to shape functionality of the optimized web application.

### Technical requirements

Previously described quality control software, SMACK, had been implemented as a command line application in Python (http://www.python.org), with no dependencies outside of the standard library.^10^ Input data is provided as an XML format file exported from Waters TargetLynx software. The outputs of the software are 2 CSV-formatted reports, one containing assay results and the other a detailed report of the quality control calculations. Supplemental Fig. 1 displays the flow chart defining the quality control algorithm, which has been simplified since its initial implementation. For this project, we needed to incorporate the software into the application to perform the same quality control calculations on the opiate assay raw data and then display the output to the user in a meaningful manner. Moreover, our group has a defined technical software stack for developing and deploying applications in Python. For these reasons, writing the application in Python was the most efficient choice. An additional requirement was to automatically file results into the LIS (Clinisys Information Systems ©). To achieve this requirement, we had to work with Data Innovations (DI) Instrument Manager.

### Performance measurement and user feedback

Finally, to assess the performance, we surveyed stakeholders and documented the rough amount of time that is required in each step of the workflow. Input was solicited from several laboratory staff members rotating through the area regarding how much time was spent on each step before and after the implementation of the application. We also surveyed the pathologists in training (chemistry fellows and rotating residents), known as trainees. Finally, we extracted data from the LIS to capture the number of occurences in which a testing order had a correction or modification after the initial data entry so we could compare correction rates prior to and after implementation.

## Results

### Laboratory workflows prior to implementation

Providers order UDTs for the patient based on an assessment of the patient's risk for compliance. Table 1 displays the patient risk levels, the intended population, and the tests associated with each risk panel.

**Table 1:** Urine drug testing ordering strategy based on risk. Providers assess risk, review clinical patient care data, and order tests as described.

Risk	Intended population	Tests
Low	Morphine equivalent dose <90 mg/day; Stable on chronic opioid therapy or buprenorphine therapy	Drug Screen Immunoassay
Medium to High	Morphine equivalent dose >=90 mg/day; Aberrant behavior (including early prescriptions, lost prescriptions, outbursts in office)	Drug Screen Immunoassay; Enzymatic Assay for alcohol; Opiate Confirmation LCMS
High	Same as medium to high; added concern for clonazepam or lorazepam	Drug Screen Immunoassay; Enzymatic Assay for alcohol; Opiate Confirmation LCMS; Benzodiazepine Confirmation LCMS

For each panel, providers have the option to enter any drug the patient is expected to be taking which may influence the test results. Each test in the panel is conducted separately and the results are entered into the LIS. Once all the tests have been completed and results entered, the pathologist may begin generating an interpretation.

When pathologists are reviewing the results, they consider the patient's history in the electronic health records (EHR) for information that may influence the test results such as medications not specified by the ordering provider. These notes would be entered in dedicated fields in the spreadsheet. For training purposes, a pathologist trainee will often review the results first, then review their work with a director who may discuss the case, edit the preliminary interpretation, then make the final approval. Upon review of cases that initially only received the immunoassay drug screen, a probable outcome is for the reviewing pathologist to deem additional confirmation testing necessary. Therefore, the workflow is the summation of 2 separate parts, cases which receive opiate confirmation testing versus those that do not. The consideration to separate the workflow into 2 parts was partly because many orders require additional reflexive orders which influences how quickly the final interpretations will be released. For this reason, it was best to group cases by risk level to provide an interpretation as quickly as possible. Laboratory staff worked with departmental software engineers to create and deliver spreadsheets for each workflow which contain the patient's demographics (medical record number, age, sex, name, and order number) along with the results of the tests.

The opiate assay is performed on a Waters Xevo TQ-MS tandem mass spectrometer with an Aquity UPLC system. Data acquisition is controlled by Waters MassLynx and chromatography review is completed using Waters TargetLynx software. When the data acquisition is complete, the technologist completes a cursory review of the auto-integration using TargetLynx. Once the preliminary review is complete, an XML file with the raw data is created. The XML file is the input of the application that performs quality control calculations from the LC-MS/MS raw data. The output was 2 detailed tabular reports; the first was the calculated results after the algorithm was applied and another with a detailed report of the quality control calculations. These reports would be reviewed in Microsoft Excel by 2 laboratory staff members to address any values that did not meet quality control standards before entering the results into the LIS. Once the results have been reviewed, staff would send an email to the reviewing pathologists that reports were ready for their review. The pathologist would then combine the calculated results with the spreadsheet containing other test results and patient demographics via a copy/paste method. During the pathologist review, they may identify aberrant results stemming from interfering compounds or possible adulterations based on the data. They would then create an email thread with the laboratory to communicate and resolve the issue.

One possible outcome for those cases where patient risk levels are associated with low to medium risk, a reviewing pathologist orders a confirmatory test. Pathologists would type the test codes for the orders they wished to place in the spreadsheet and the reviewing MLS would place the orders in the LIS. Therefore, a third spreadsheet was created to capture the results for the cases that received additional tests. To transfer the notes captured during the initial review, a Microsoft Excel macro was created which would parse the spreadsheets and bring the pathologist's saved work from the initial spreadsheet into the final spreadsheet.

These spreadsheets were created and delivered via a dedicated website where staff and faculty were able to download the spreadsheets from any computer in the laboratory. After the spreadsheet was initially downloaded and edited, the file was stored on a shared drive accessible through a protected network that was accessible to the laboratory and pathologists.

A naming convention for these files was created to help indicate the stage in the workflow.

Once the reviewing pathologist had completed their review, they would send an email to staff so that they could enter the interpretation result into the LIS manually. If the technologist identified any issues with the interpretation, they would email the pathologist to edit/review the interpretation.

### Technical requirements

The application was implemented in Python using the Flask web framework (https://flask.palletsprojects.com/) and other open-source libraries. Supplemental Table 1 lists the required libraries used for the application.

The software was developed on an Apple Macintosh running Mac OS 10.15 and is used in production on Amazon Web Services (AWS) server running Ubuntu (18.04 LTS). The data from the LIS (Clinisys Information Systems ©) is gathered using Cache Object Script that collects information every 30 min from 6am to 6pm and is delivered to a secure cloud storage resource (S3 bucket) on AWS. Once the results are finalized, the application sends a tab-delimited file to a transfer Linux (4.15.0) server which is picked up by Data Innovations instrument manager that sends the data to the LIS.

An important component of the software development and deployment process was the use of the Git version control software. Git is a free, open-source, distributed version control software that can be used to store “snapshots” of source code files in which modifications are captured to the code repository. Each change is attributed to a specific author and can be associated with a comment describing the intent of the change. A unique tag identifies each change, and the application captures the tag in the software version number and records this identifier to every data output. In this manner, results of each analysis can be traced to the exact version of the software used to collect and generate those results. Additionally, GitLab is a Git-based fully integrated platform which allows users to create “issues” where enhancements, development, and other topics may be discussed collectively. This feature of GitLab can also attribute labels to each issue so that larger topics or concepts can be collectively viewed and labeled.

The application is a web-based application that can be launched from any web browser. Access control is managed using our institutional identity provider with single sign-on (SSO) with 2 factor authentication. For auto-resulting, once the results reach their terminal status (pathologists and laboratory staff have completed their collective review), a tab-separated-values (TSV) is sent to the Data Innovations Instrument Manager where it files the results to the LIS.

### Requirements

The data collected and workflows described serviced as the foundation of the application's design. The application needed to gather the necessary data from the LIS and display the information in a manner that is useful to the user. A system needed to be designed to progress a case through the necessary steps for the appropriate reviewer. Entry fields needed to be available for all members to be able to communicate problems, capture notes by the pathologists during their review, and for the final interpretation result. The application needed to ingest the XML file from TargetLynx of the opiate test, apply the SMACK algorithm, display both the QC calculations and results, and save all edits. Lastly, the application needed a system to communicate any additional confirmation tests the pathologist wanted to order.

A new component within the laboratory workflow was the introduction of an automated liquid handler to prepare the urine samples for the opiate assay. The laboratory had been manually preparing the samples and entering the patient identifiers into the acquisition system, MassLynx. When creating the extraction method of the liquid handler, the decision was made to use the barcode on the specimen label as the sample identifier for the assay. To associate the result back to the patient, all specimen IDs also needed to be gathered.

**Fig. 1:**
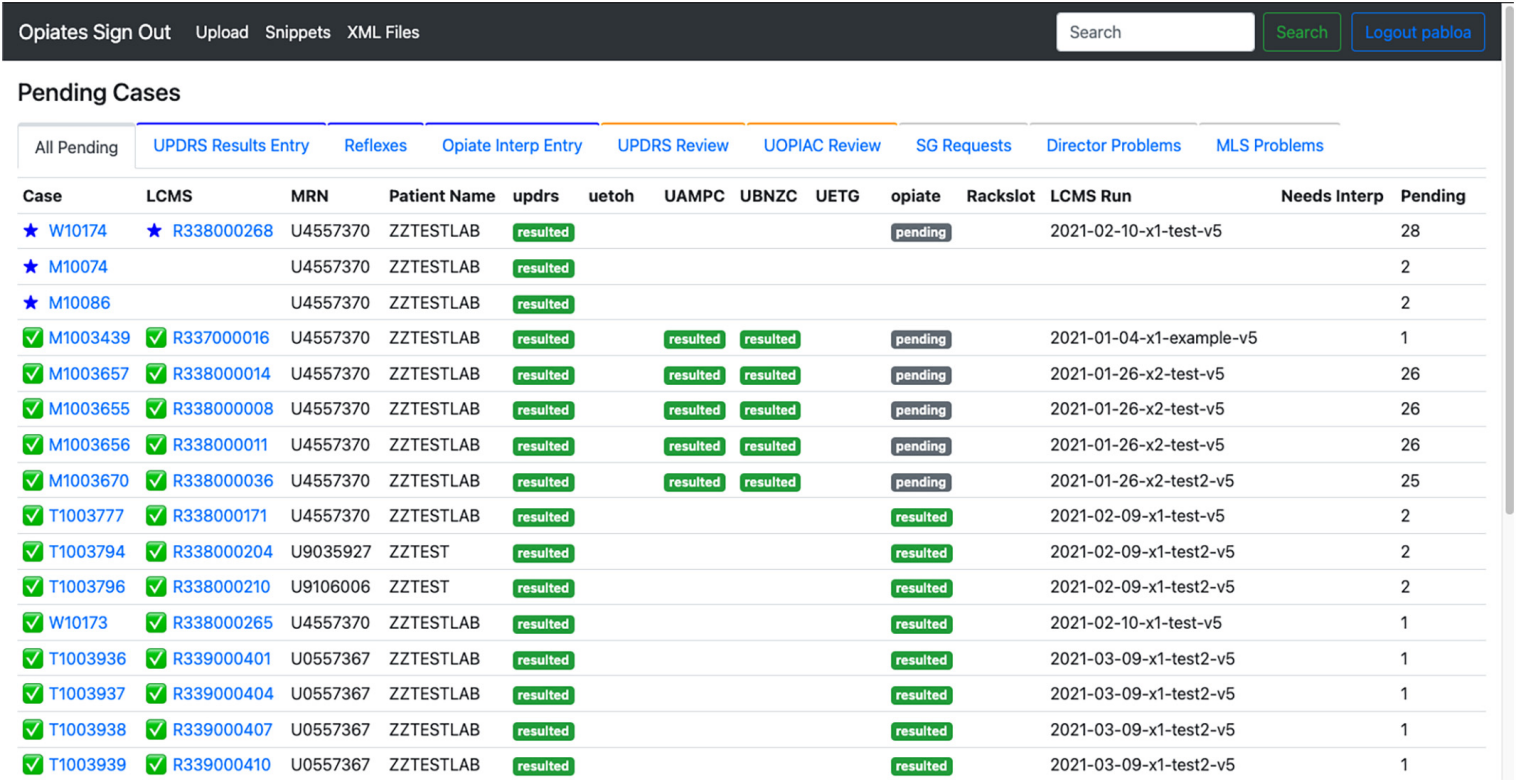
Home page of the opiate sign-out application. The landing page lists all pending samples in the application along with sample information such as case number, container ID (LCMS), medical record number, patient name, and status of each test.

The decision to create a web-based application was to provide an easy-access entry point for the laboratory and pathologists. Also, a web-based application provided the flexibility needed to create a custom site that fit the needs of the workflow. Fig. 1 shows the home page of the application.

**Fig. 2:**
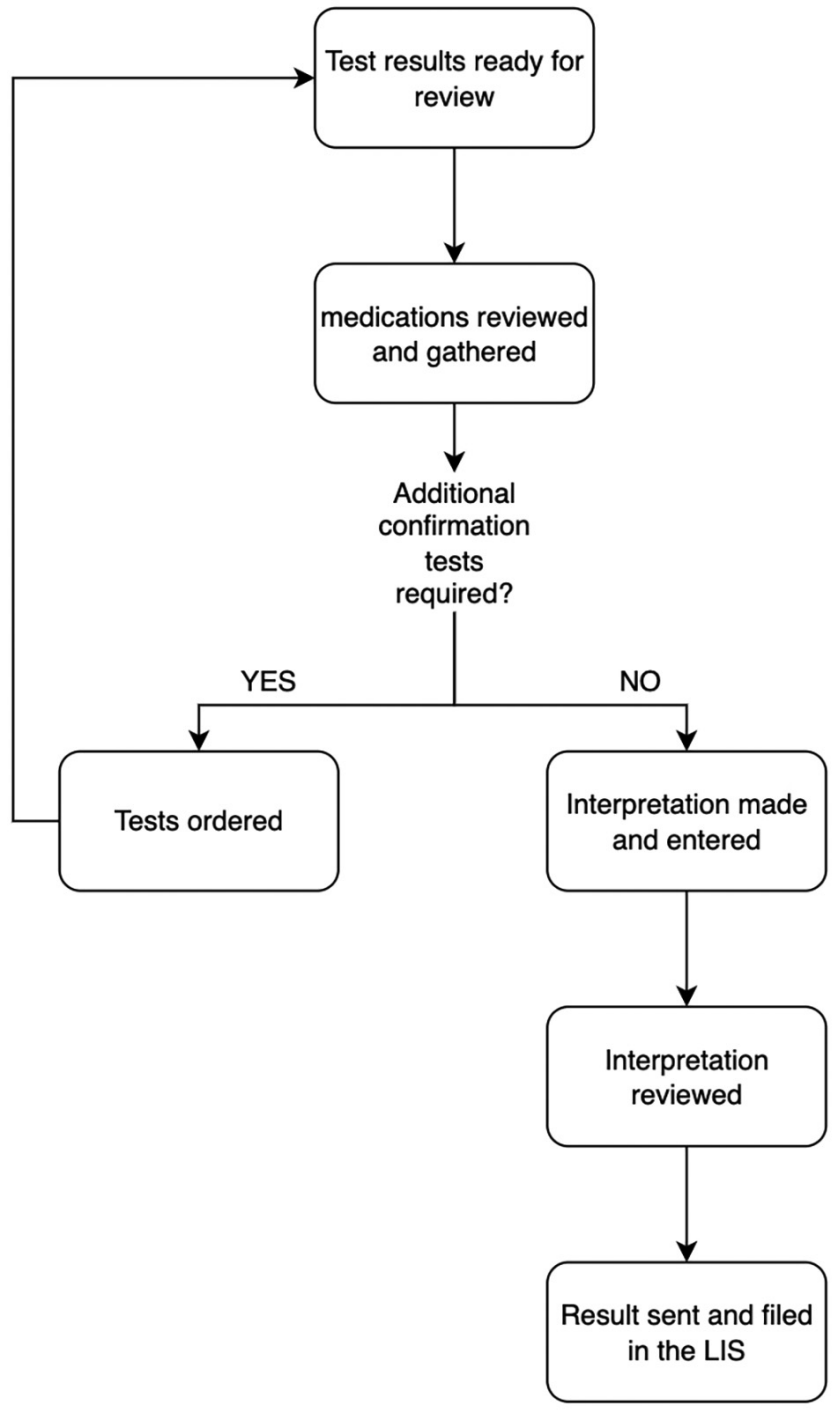
General workflow outline. The status of each case drives the case from one step in the workflow to the next until the result is filed into the LIS.

We provided a tabular pending list that indicates the case status, order status, order number, LCMS results page, medical record number, patient name, and the status of each test. Cases were provided status IDs to progress each case through the different steps of the workflow. While investigating the overall workflows we determined that workflows could be categorized by the different stakeholders, the pathologists and the MLSs, and the different tasks associated with each personnel. From this, we created filters for the pending lists so that each user could easily jump into their associated workflow and collectively work on cases based on the case status (Fig. 2). We added a search bar so that a user could search any pending or historical case by name, order number, hospital ID, or specimen ID.

**Fig. 3:**
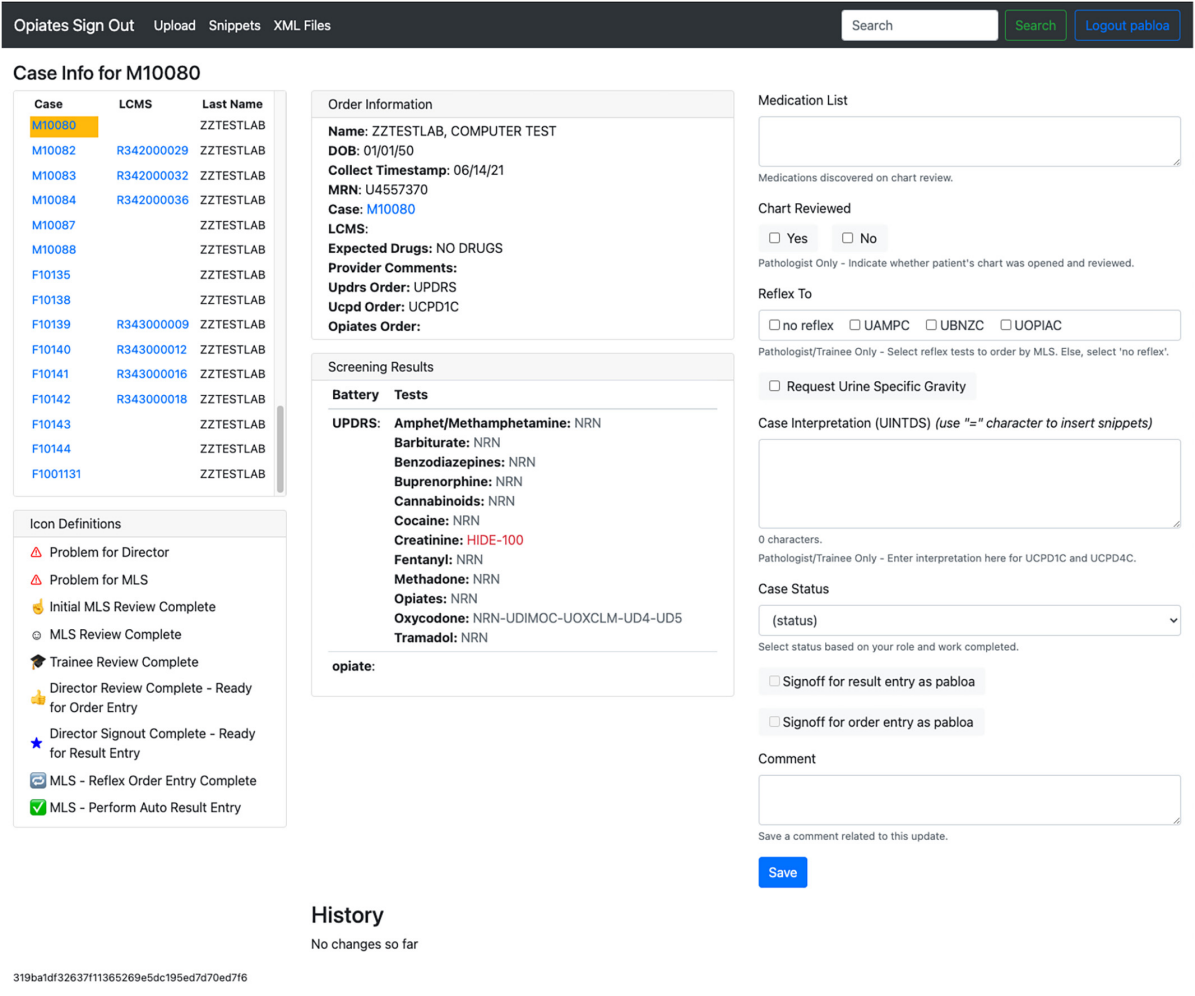
Case page example. The page displays the fields and layout of the case page of a given patient.

For each case, a new page is created where the patient demographics, test results, and entry fields are displayed. Fig. 3 shows what the case page looks like in the application for a given patient and Table 2 lists the separate features displayed on the page.

**Table 2:** Listed case page features. Each listed feature is an object that supports the process of generating the test interpretation by a pathologist.

No.	Feature	Description
1	Pending case list	A list of all the pending cases for review.
2	Icon definitions	The key maps out the status symbols and their definitions.
3	Order information	Patient and order information of the overall case.
4	Screening results	This card displays the results of the tests by test component. If a test is still pending or incomplete in the LIS, a gray “pending” box will be displayed in the “Tests” column.
5	Medication list	Entry field where medications found in the patient’s chart are entered.
6	Chart reviewed	For billing purposes, pathologists indicate whether the patient’s chart was reviewed during review. A value that is entered as a result into the LIS.
7	Reflex to	Pathologist will mark these boxes to indicate which additional tests will be ordered by the MLS in the LIS.
8	Request urine specific gravity	Pathologist will check this box for MLSs to perform a specific gravity test and they will populate the entry box with the result.
9	Case interpretation (UINTDS)	Entry field used to capture the pathologist generated interpretation.
10	Case status	Dropdown box used to communicate and move the case through the sign-out process. Statuses are changed after the person has completed their duties for the case.
11	Signoff check boxes	When a director checks this box, it will gather their name and associate it with the additional order or result for billing purposes.
12	Comment	Entry field used to gather comments, questions, concerns regarding the overall case.
13	Save	This button will save all input and changes made to the page.
14	History	Historical changes and comments are displayed with the employee ID of the person who made the change and timestamp of the change.

**Fig. 4:**
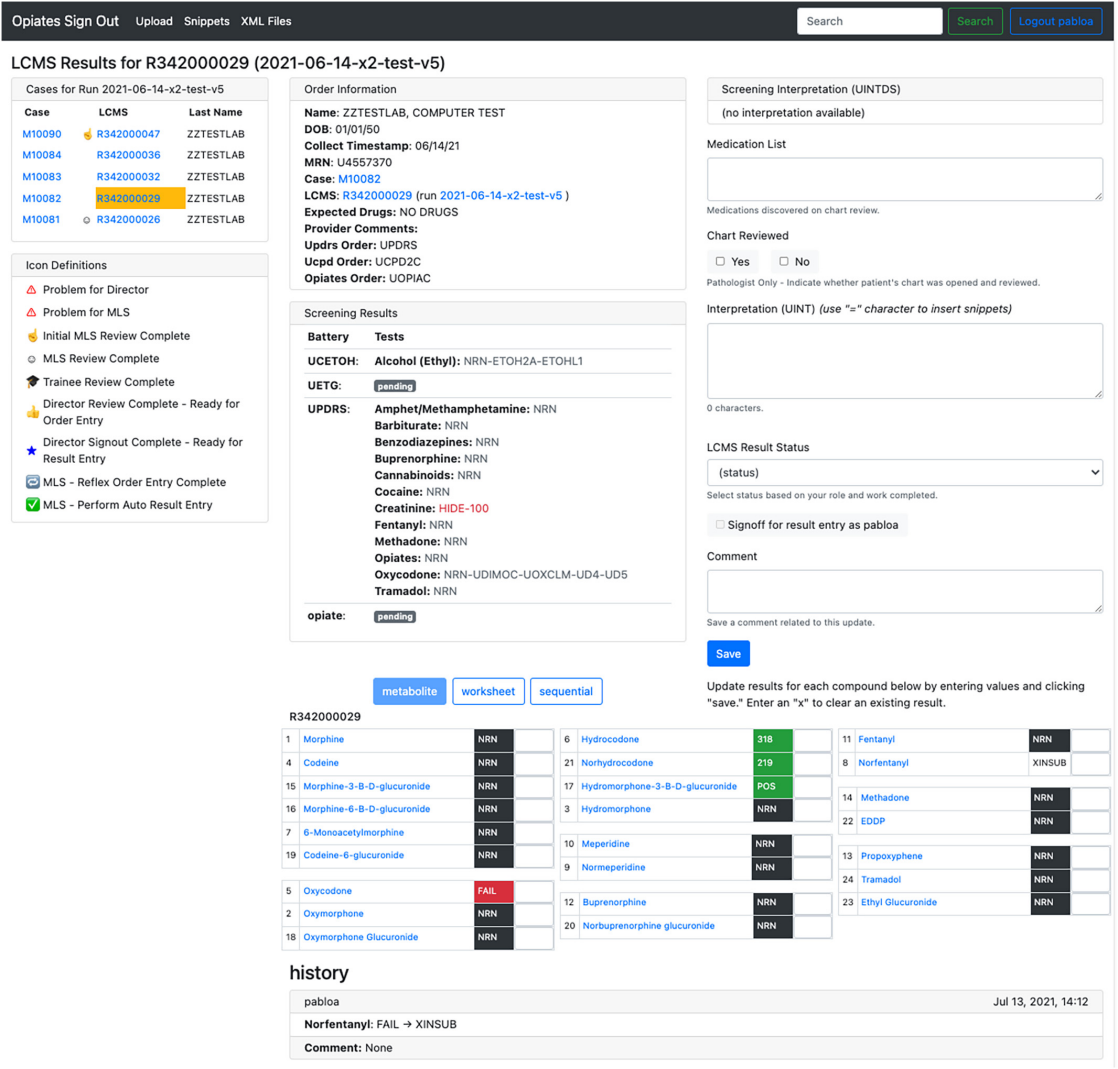
LCMS page example. The figure displays the several features of the LCMS page for a given patient including the output table of the SMACK quality control calculations.

For orders where the opiate LCMS confirmation test was placed, a separate page is created known as the LCMS page. Fig. 4 displays the LCMS page for a given patient. This page holds much of the same information as the case page but with dedicated space for the result of the quality control calculations. In this manner, we avoid cluttering a single page and if a case did not have the opiate confirmation test ordered the page only shows relevant information. When the LCMS page exists, the specimen ID is the basis of the URL of the LCMS page. This page duplicates many of the features displayed on the case page to help highlight key information and ease of access to resources for the reviewing pathologists.

When an XML file is uploaded to the application, the application applies the algorithm and automatically associates the result with a patient using the unique specimen ID. The result table can be viewed in 1 of 3 configurations.

1. Metabolite – compounds are listed together by metabolic groups.
2. Worksheet – compounds are listed in the order seen in the LIS.
3. Sequential – compounds are listed in the order seen in TargetLynx.

The opiate confirmation assay is quantitative/qualitative. Values that require review by the laboratory staff for QC purposes are highlighted in red. Positive values are highlighted in green. Either a positive numerical value is displayed for quantitative results or “POS” noting positive is displayed for positive qualitative results. Negatives are displayed in black and denoted by “NRN”.

Once the interpretations have been created, the laboratory staff will review it for accuracy (ensuring that each drug/analyte is mentioned) before submitting it to the LIS. If they identify a problem, they will change the status of the case to “Problem for Director”. This status captures all cases that requires special attention by the attending pathologist also known as Director. Once the problem has been addressed, the pathologist may change the status to the appropriate selection.

The status has been the main factor for progressing a case through the workflow. We recognized that the overall workflow could be split by personnel, the MLS staff, and pathologists, and could be further broken up by the steps in those workflows. Therefore, we developed separate tabular views (Table 3) that collectively group pending cases based on the step in the workflow the case currently resides.

**Table 3:** Tabular views of pending cases. Each tab holds a list of cases based on the state the case resides in the workflow.

No.	Table Filter	Description
1	UPDRS results entry	For these cases, the immunoassay results have been reviewed and an interpretation has been finalized by an attending pathologist. The interpretation of these cases requires a review by an MLS to ensure that there are no expected compounds or medications that were not mentioned in the interpretation.
2	Reflexes	This is a list of cases that have been reviewed by an attending pathologist and have been requested/approved to order a reflex test by an MLS in the LIS.
3	Opiate interp entry	For these cases, the opiate assay results have been reviewed and an interpretation has been finalized by an attending pathologist. The interpretation of these cases requires a review by an MLS to ensure that there are no expected compounds or medications that were not mentioned in the interpretation.
4	SG requests	This tab holds a list of cases which have been marked by an attending pathologist for a request to MLSs to conduct a Specific Gravity test. This test will not be billed or appear in the LIS.
5	MLS problems	These cases have been marked as “Problem for MLS” by a trainee or attending. The comment section will indicate what the problem pertains. These cases require a review by MLSs.
6	UPDRS review	These cases require a review of the immunoassay results and medications for the patient to be collected. Once complete, a decision to either order reflex tests or make an interpretation needs to be made.
7	UOPIAC review	These cases require a review of the final opiate LCMS confirmation assay results and medications for the patient to be collected, potentially, based on the initial order. Once complete, a decision to either order reflex tests or make an interpretation needs to be made.
8	Director problem	These cases have been marked as “Problem for Director” by a trainee or MLS. The comment section will indicate what the problem pertains. These cases require an additional review or resolution by the attending pathologist(s).

### Performance measurement and user feedback

Several new cases are ordered every day. In 2020, there were roughly 150 interpretations generated per week. Having dedicated queues for each step in the workflow helped staff and pathologist coordinate the work. Also, the queues helped pathologists prioritize and schedule the work based on the amount of time each pathologist had at a given time.

To investigate the return on investment, we surveyed the users on the amount of time each step in the workflow took to complete both before and after the application. The workflow no longer involved worksheets. Before the application, users reported they often spent 1–2 h per day managing and combining files. This number came down to zero as they no longer had to use worksheets. Additionally, the introduction of the liquid handler to automatically prepare samples for the assay helped gain back an hour of time the MLS spent preparing the samples manually. The greatest return for staff was moving from manual-result-entry to auto-result entry. The test is conducted 5 times per week and requires 6 days of staffing to process and manage data. Each batch requires the review of 2 laboratory staff members and the practice continues but they would manually enter the results into the LIS. Staff reported they would spend around 1 h per batch entering the results. This number came down to virtually zero as the result entry was automated via the flat file transfer to Data Innovations. For pathologists, they reported the interface was easier to navigate the multitude of values in a spreadsheet. The organization helped keep better track of cases and organize the workflow to be more efficient. On average, pathologists reported they have gained back 2 h per batch since the application has been incorporated into the workflow. MLS staff reported they gained back 4 h per batch since the application has been incorporated into the workflow.

In the 24 months prior to implementation of the application, there were 24 256 total opiate mass spectrometry test orders and 449 orders had at least 1 data entry modification (defined as at least 1 test component having a result entered more than once), for a modification rate of 1.9%. After implementation, over 21 months there were 18 112 total opiate orders and only 10 orders had at least 1 data entry modification, yielding a modification rate of 0.06%.

## Discussion

We have developed an application that centralizes data for UDT interpretation. Moreover, the automatic aggregation of this data provides an efficient and convenient method for pathologists to review the data to generate an interpretation of the results for clinicians and providers to review with their patient's undergoing opioid therapy. There are various groups that have developed novel web-based applications to help increase efficiency.^11^, ^12^ Groups have reported that their applications have reduced errors, expenses, staff burnout, and increased the level of quality patient care. Laboratory staff were able to gain back several hours from what they would have spent manually entering results and managing spreadsheets. After the application was introduced, the staff scheduled to the opiate bench was reduced from 2 full-time employees, 6 days a week to 1 full-time employee, 6 days a week. Often the reviewing laboratory staff has worked in the department for some time before training for this work. Freeing an experienced laboratory member's time allowed them to help in other critical areas in the laboratory. Additionally, since the transfer process was fully automated, it reduced the number of errors transmitted into the LIS and time spent contacting the provider who may have already reviewed the results. In Mays et al,^13^ the authors found a 3.7% error rate of manual transcription versus auto-entry. In our analysis, 1.8% of orders required a modification prior to implementation and post-implementation, this rate was reduced significantly to 0.05%. While it is likely that some modifications were made within the application as multiple team members composed and reviewed results prior to release, misinterpretation or confusion could be avoided by preventing information that needed modification from being entered into the LIS and seen by the provider or patient.

The informatics team dedicated substantial effort through a year and a half timeframe to develop and implement the application. The team included 1 member responsible for interviewing the stakeholders and documenting the requirements, a faculty member who designed the software architecture and database schema and developed the application prototype and deployment infrastructure, 2 software developers who worked together to complete the application, 1 software developer who helped interface the LIS to gather the necessary patient and order information, and 1 LIS specialist to help configure the LIS for auto-filing the results. Team members had specific tasks which could have been worked upon in parallel, although many tasks relied on the completion of a task by a separate team member. Additionally, no member dedicated their time solely to this project during the project span. Each member balanced their time across several projects. The overall estimated time spent by the team was roughly 350 h over 18 months. The application was developed by departmental programming staff, one of whom was hired specifically to support the Chemistry division, while the others were members of a departmental pool of informatics resources. Having a departmental informatics team that can develop novel solutions to complex, clinical problems require considerable resources but there are also a variety of opportunities across units of the laboratory that benefit from high efficiency.

After its initial launch, updates and enhancements were made to the application. The enhancements included bug fixes and additional features that were requested from users after working with the application for some time. The amount of time was roughly 130 h over 18 months. The amount of time spent in developing and enhancing the application was significant. However, introducing the application to the workflow freed up a full-time laboratory employee’s time. Moreover, the laboratory has been able to increase the sample volume with no increased need in MLS staffing.

The return on investment for the laboratory was significant. We demonstrated that the primary benefit to implementing a custom application has been a profound improvement in workflow and staff efficiency. The application removed mundane and repetitive tasks such as transferring data from one spreadsheet to the another. Additionally, it also removed the need for laboratory staff to fully complete the batch review before a reviewing pathologist could begin their review. With the application, the pathologist could begin as soon as the first patient in the batch was reviewed by the laboratory staff member. Additionally, the process of auto-filing results could be extended to other tests within the department, freeing some additional time for staff. This provides evidence to the value of developing and implementing custom applications.

A consideration that groups should make when developing custom applications is the time required to understand the problem. For this project, we spent several hours interviewing stakeholders to understand the workflow and the different needs of each personnel. Once requirements were gathered, a plan needed to be discussed and agreed upon by members of both the Informatics and Chemistry departments. Another challenge groups should consider is the potential requirement to rewrite software programs and repeat validation with upgrades to the software on top of any tests needed to be conducted to ensure the integrity and accuracy of a custom application. For the initial validation of the application, the informatics and chemistry teams discussed and agreed upon a validation plan. The plan involved laboratory members testing the application’s functionality by working through the application workflow with test cases and matching the tested output with expected output. Additionally, groups should consider the availability of the development team to support their custom application. The need for support could stem from latency of servers due to traffic, system downtime, or undiscovered bugs that affect the behavior of the application. For these reasons, a support plan is recommended that is distributed to both users and supporting staff so that users know who to contact and IT staff understand how to effectively address the problem. The support plan may also require that laboratory staff keep up to date with manual result entry processes in case electronic systems undergoes an unexpected downtime for a prolonged period. Lastly, the need to be cognizant of PHI-related IT security increases the need of IT involvement for the implementation and ongoing support of custom applications.

In our laboratory, the opiate LCMS confirmation assay is one of the most labor intensive assays because of the sample preparation method and data review steps. This work laid the foundation to move other LCMS assays to automation. As this trend continues, our staff will be able to spend their time reviewing critical issues and tasks, further increasing the efficiency in the laboratory.

## Declaration of interests

The authors declare that they have no known competing financial interests or personal relationships that could have appeared to influence the work reported in this paper.

## Appendix A Supplementary data

Supplementary material Image 1
