# Supplementary material for: A web application to support the coordination of reflexive, interpretative toxicology testing
Source: J Pathol Inform. 2023 Feb 26;14:100303. doi: 10.1016/j.jpi.2023.100303 (PMC10024164; doi:10.1016/j.jpi.2023.100303)
Supplement: Supplementary file 1 — Supplementary material [file mmc1.docx]

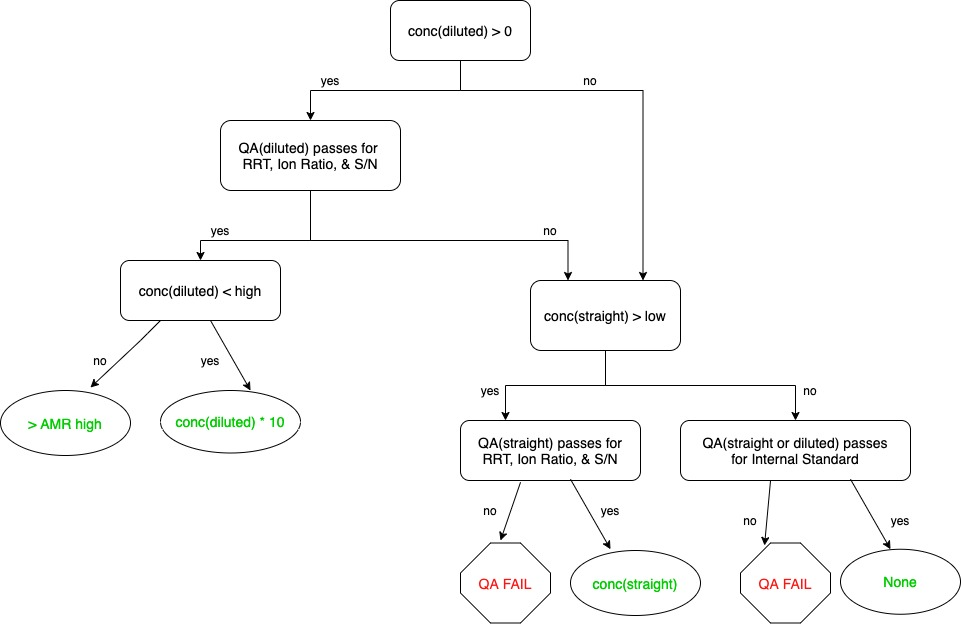


**Supplemental Figure 1. Flow chart defining the quality control algorithm.** The quality control (QC) calculations consider the relative retention time (RRT), ion ratio, signal-to-noise ratio (S/N), internal standard signal, and calculated concentrations of the compounds for the sample. Also, the concentration relative to the upper (high) and lower (low) bound of the analytical measurement range (AMR).

| Library | Version |
| --- | --- |
| gunicorn | 19.9.0 |
| Jinja2 | 2.10.1 |
| Flask | 1.1.1 |
| Flask-WTF | 0.14.3 |
| Flask-RESTful | 0.3.7 |
| lxml | 4.4.1 |
| boto3 | 1.9.242 |
| botocore | 1.12.242 |
| s3fs | 0.3.4 |
| pip-api |  |
| psycopg2-binary | 2.8.4 |
| Markdown | 3.2.2 |
| paramiko | 2.7.2 |
| pandas | 1.1.2 |

**Supplemental Table 1. Python Libraries for Application.** The table lists the open-source libraries included as direct dependencies of the application along with the versions used.
